# Amphiphilic DNA nanostructures for bottom-up synthetic biology

**DOI:** 10.1039/d1cc04311k

**Published:** 2021-10-28

**Authors:** Roger Rubio-Sánchez, Giacomo Fabrini, Pietro Cicuta, Lorenzo Di Michele

**Affiliations:** Biological and Soft Systems, Cavendish Laboratory, University of Cambridge JJ Thomson Avenue Cambridge CB3 0HE UK l.di-michele@imperial.ac.uk; fabriCELL, Molecular Sciences Research Hub, Imperial College London London W12 0BZ UK; Department of Chemistry, Molecular Sciences Research Hub, Imperial College London London W12 0BZ UK

## Abstract

DNA nanotechnology enables the construction of sophisticated biomimetic nanomachines that are increasingly central to the growing efforts of creating complex cell-like entities from the bottom-up. DNA nanostructures have been proposed as both structural and functional elements of these artificial cells, and in many instances are decorated with hydrophobic moieties to enable interfacing with synthetic lipid bilayers or regulating bulk self-organisation. In this feature article we review recent efforts to design biomimetic membrane-anchored DNA nanostructures capable of imparting complex functionalities to cell-like objects, such as regulated adhesion, tissue formation, communication and transport. We then discuss the ability of hydrophobic modifications to enable the self-assembly of DNA-based nanostructured frameworks with prescribed morphology and functionality, and explore the relevance of these novel materials for artificial cell science and beyond. Finally, we comment on the yet mostly unexpressed potential of amphiphilic DNA-nanotechnology as a complete toolbox for bottom-up synthetic biology – a figurative and literal scaffold upon which the next generation of synthetic cells could be built.

## Abstract 2

Here we review our recent efforts, and those of others, on the construction of biomimetic DNA nanostructures that imitate biological structures and functionalities, and could serve as a generalised platform for engineering artificial cellular systems.

## Graphical abstract

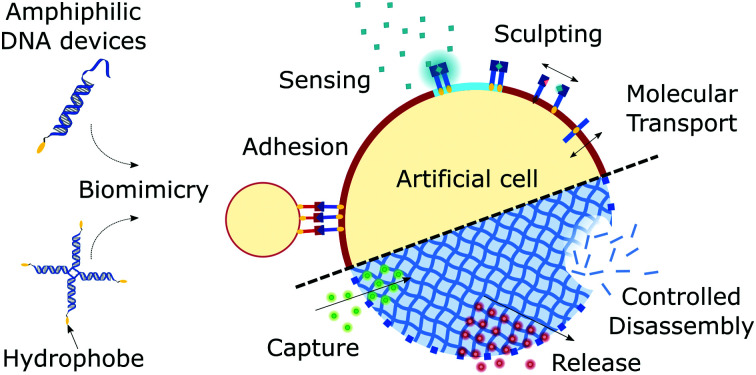

## 1 Introduction

Since its advent in the eighties,^1^ DNA nanotechnology has evolved from an inspiring concept to a practical toolkit driving advances in several areas of fundamental and applied science.^2^ Owing to the programmability and selectivity of the Watson–Crick base-pairing, DNA motifs have been widely employed to experimentally explore the self-assembly phenomenology of macromolecular,^3^ colloidal^4–7^ and nanoparticle systems.^8–10^ By affording unprecedented control over the shape,^11^ interactions,^12,13^ and mechanical properties of nanostructures,^14^ the DNA origami technology has opened further new avenues for the field, with concrete applications to biomolecular scaffolding,^15–17^ single-molecule analysis,^18,19^ biosensing,^20^ nanomedicine,^21^ imaging,^22^ and the construction of advanced materials.^23,24^ In parallel to structural control, our growing understanding of nucleic acid kinetics and thermodynamics has resulted in the ability to program dynamic responses,^25^ marking the advent of DNA-based molecular computing^26–28^ and the development of proof-of-concept actuable nanodevices,^29,30^ biosensors,^31,32^ and technologies for optical imaging.^33–37^

Uniquely among artificial molecular-scale devices, the sheer variety of architectures and responses demonstrated with nucleic-acid nanostructures makes them comparable with biologically occurring machinery in terms of complexity and functionality.^38–41^ While early incarnations of DNA nanotechnology drew inspiration from the simplest biochemistry and biological nanomachines, efforts in the field now aim to match and often surpass what biological evolution has produced.^42,43^

It has therefore become apparent that DNA nanotechnology holds great potential as a toolkit for engineering all aspects of man-made mimics of biological cells – the central objective of the rapidly expanding discipline of bottom-up synthetic biology. The biomimetic microrobots, often referred to as artificial cells, are constructed from inanimate molecular components to replicate emergent responses typically observed in biological cells, including energy conversion, communication, motion, adaptation and, although yet unachieved, replication and evolution.^44–46^ Besides their use as minimalistic models for unravelling the principles of life and probing pathways for its emergence,^47,48^ artificial cells are expected to revolutionise numerous emerging technologies, from biosynthesis to materials and healthcare. In the latter context, notably, artificial cells are envisaged as the basis of next-generation personalised therapeutic solutions where, operating *in vivo*, the microrobots could sense disease-related biomarkers, record their presence, and respond with the *in situ* synthesis and delivery of therapeutic agents to reduce toxicity and boost the efficacy of treatments.^49–51^ While still far from the self-sustaining complexity of their biological counterparts, artificial cell technologies carry several potential advantages compared to mainstream platforms based on engineered biological cells, including a greater freedom of design and choice of materials, compatibility with non-biological components, efficient use of resources and possible avoidance of redundancies, limited biological risks, and less stringent regulatory constraints.

The construction of cell-like objects requires control over (at least) two distinct, yet intertwined aspects: compartmentalisation and information processing. The former is needed to separate the synthetic cell from its surroundings and establish the internal chemical heterogeneity required to sustain synthetic molecular pathways. Several materials, from colloids^52,53^ to polymers^54^ to proteins^27,55^ have been adopted to build semi-permeable compartments for artificial cells, but the most common implementations rely on lipid bilayer membranes owing to their facile production, bio-compatibility and general similarity with cell membranes.^56^ Membrane-less compartments in the form of hydrogels^57–59^ and polymer coacervates^60–63^ represent a robust and increasingly popular alternative, mimicking similar environments found in biological cells.^64–67^ Information processing, broadly including environmental/molecular sensing, signal transduction, communication, and information storage/replication/propagation, is in most cases sustained by reconstituted biological machinery such as receptors, enzymes, ion-gating and pumping channels, and cell-free transcription/translation systems.^44,46,68,69^ In view of the ubiquitous presence of lipid interfaces, membrane-bound protein machinery is often featured in artificial cellular systems, regulating transport, adhesion and morphological responses.^69–73^

In this feature article we delve into several examples of synthetic DNA nanostructures designed to replicate the structure and functionality of biological molecular machinery in the context of cell mimicry, focusing in particular on bilayer-anchored devices acting as synthetic receptors mediating adhesion,^74–80^ tissue formation,^74,81^ communication^82^ and membrane sculpting.^83–87^ Attachment of the DNA nanostructures to lipid bilayers often requires their modification with hydrophobic moieties, such as cholesterol, tocopherol or lipids, giving the nanostructures an amphiphilic character.^88^ Besides enabling membrane anchoring, the latter can also be exploited to enhance the self-assembly capabilities of DNA nanostructures in the bulk. In the second part of this feature article we review recent findings on the construction of nanoporous networks from amphiphilic DNA motifs with well controlled structure and functionality, which could form the basis of membrane-less compartments for artificial cells and, more broadly, impact biotechnology and therapeutics.^89–91^ Finally, we discuss the broader implications of (amphiphilic) DNA nanotechnology as a versatile and integrated toolkit to simultaneously prescribe structural and information processing aspects in synthetic cells, identify key outstanding challenges, and express our view on those functionalities that could have the greatest fundamental and technological impact.

## 2 Programming biomimetic responses with membrane-associated DNA nanodevices

**Fig. 1:**
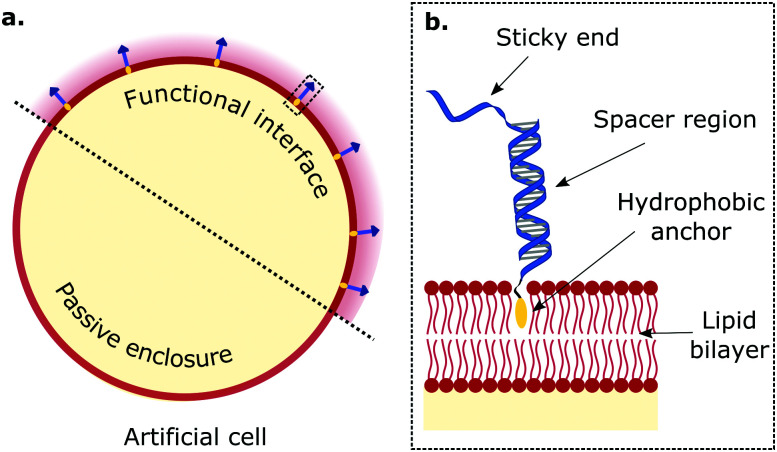
Biomimetic DNA receptors as functional platforms hosted in synthetic interfaces of artificial cells. The tools of DNA nanotechnology enable engineering functional interfaces with biomimetic DNA devices designed to replicate key features of biological membranes. (a) Amphiphilic DNA nanostructures, bearing a hydrophobic motif, decorate lipid membranes by inserting and secluding the hydrophobe in the bilayer's core. (b) Zoom-in of region enclosed in dashed rectangle in panel (a). Generally, the anatomy of membrane-anchored DNA “receptors” consists of a hydrophobic anchoring agent (*e.g.* cholesterol, tocopherol, lipids, porphyrin), a (single or double-stranded) spacer region, and a sticky-end. The latter is able to mediate interactions with other DNA receptors through sequence-based complementarity, and impart functional traits to artificial cells.

Artificial cellular technologies are reliant on compartmentalised microenvironments to promote and sustain (bio)chemical pathways. Synthetic lipid bilayers are frequently used to construct enclosures for artificial cells, but they usually lack the functionalities central to their biological counterparts. Therefore, engineering synthetic lipid interfaces with increasingly diverse functionalities represents a necessary stepping stone in artificial cell science (Fig. 1a). While most approaches for establishing responsiveness in artificial lipid bilayers have relied on the reconstitution of protein-based machinery,^69,72,73,92–94^ the programmability afforded by DNA nanotechnology offers an increasingly popular alternative.^2^ For this purpose, synthetic DNA constructs are often chemically modified with hydrophobic motifs (*e.g.* cholesterol, tocopherol, lipids^78,95^), which make the nanostructures amphiphilic and drive their attachment to the membrane by inserting in the bilayer's core. In this section we review examples of our work, and that of others, which have used bio-inspired amphiphilic DNA nanostructures, mimicking cell-surface receptors, to replicate functionalities exhibited by biological interfaces, such as regulated cell adhesion, tissue formation, sensing, membrane patterning and transport.

### 2.1 DNA receptors to regulate membrane adhesion and tissue formation

Multivalent interactions, that is those mediated by a large number of weakly interacting molecules, are central to several biological processes, from cell adhesion and tissue formation,^96–98^ to motility,^99,100^ synapse formation,^101,102^ endocytosis and pathogen invasion.^103–105^ In cells, these interactions are sustained by various types of membrane receptors, whose affinity for their targets (ligands), size, and mechanical properties have evolved to harness the statistical–mechanical phenomenology of multivalent interactions. A variety of synthetic membrane-anchored DNA nanostructures, hereon referred to as DNA receptors due to the similarity in the achieved functionalities, have been programmed to imitate the diverse array of properties of their biological counterparts, and thus replicate the complex phenomenology of multivalent membrane adhesion. DNA-mediated multivalent interactions between lipid bilayers have thus been designed for and studied with the dual purpose of elucidating biologically-relevant physical principles and programming adhesion and tissue formation in artificial cellular systems.^74,78,80,88^

The structure of biomimetic DNA receptors can be generally rationalised by identifying three anatomically distinct elements: an anchoring agent, a spacer region, and a single-stranded sticky-end domain, depicted schematically in Fig. 1b.^88^ While the anchor physically confines the DNA nanostructure to the membrane, the spacer region (either single or double-stranded) determines the stiffness and extent to which the construct will project away from the interface. Sticky ends are designed to selectively bind complementary nanostructures so to produce adhesion, but can also be replaced by aptamers and non-DNA moieties (*e.g.* biotin,^77^ peptides^106^) to address targets other than nucleic acids, such as proteins (*e.g.* streptavidin, antibodies) and metabolites. These membrane-bound DNA nanostructures display lateral diffusivity at rates similar to the surrounding lipids,^107–109^ which thus enables their lateral re-distribution as driven by free-energy minimisation.^88^ In what follows, we will refer to these DNA constructs either using the bio-inspired term “receptors”, or simply as “linkers”.

The emergence of bioconjugation chemistries has allowed to link DNA molecules to a variety of functional moieties,^106,110^ such as hydrophobic and amphiphilic molecules,^111–114^ thus expanding the power of DNA nanotechnology beyond the realm of base-pairing. Such strategies enabled to interface DNA constructs with lipid-based supramolecular structures to exert control over their self-assembling properties. Indeed, even though DNA-mediated interactions have been applied to program the self-assembly of Brownian objects since the 1990s,^4,5,8–10^ the extension of this concept to synthetic lipid bilayers was popularised only in the mid 2000s thanks to the contributions of Boxer, Höök and co-workers.^115–119^ In early experiments, DNA-decorated Small Unilamellar Vesicles (SUVs) were tethered to Supported Lipid Bilayers (SLBs) *via* linkers with complementary sticky-ends. This setup was harnessed to elucidate the lateral diffusivity of the DNA-tethered SUVs^115^ and the kinetics of their docking onto the SLB.^116^ Replacing SUVs with cell-size Giant Unilamellar Vesicles (GUVs), Boxer and co-workers demonstrated the generation of (multi-storey) bilayer patches, formed through the controllable adhesion and rupture of GUVs onto SLBs.^117,118^ With the same system, the authors demonstrated the occurrence of lateral phase separation between DNA linkers of different lengths, arising from the minimisation of membrane bending energy.^119^ The latter phenomenon is reminiscent of the size-induced phase separation observed between receptors involved in immune synapses.^96,101,102,120^

**Fig. 2:**
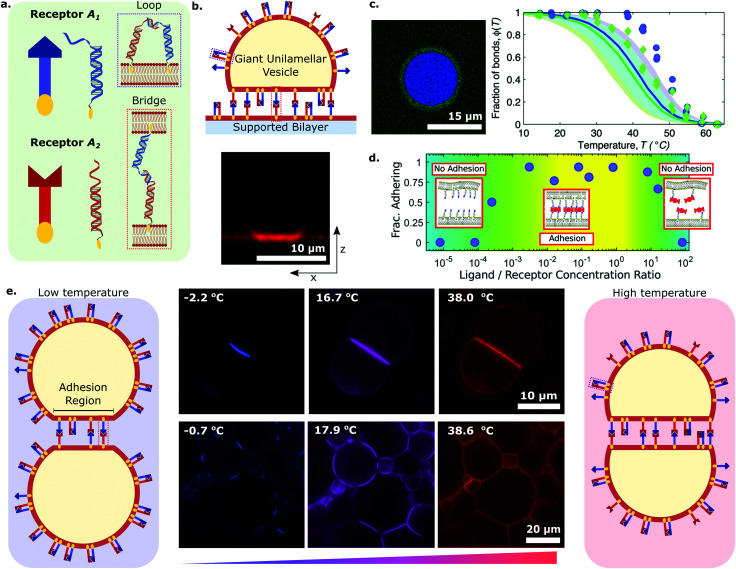
DNA receptors tune the equilibrium properties of artificial cellular networks. Synthetic DNA receptors anchored to lipid bilayer membranes *via* hydrophobic motifs can be used to control the adhesive properties of artificial cells. (a) Receptors *A*_1_ and *A*_2_ feature complementary sticky-end domains, and are present in both membranes. Therefore, they can adopt either an intra-membrane loop or an inter-membrane bridge configuration. (b) (top) Schematic depiction in which synthetic DNA receptors *A*_1_ and *A*_2_, anchored with cholesterol moieties, mediate bridging *via* 7-nucleotide sticky-ends leading to adhesion between a Giant Unilamellar Vesicle (GUV) and a Supported Lipid Bilayer (SLB). (bottom) Confocal cross-section of a tethered GUV-SLB set-up. (c) (left) Segmented confocal micrograph showing the adhesion patch between a GUV and a SLB (blue) and the surrounding supported lipid bilayer (green). (right) Experimental (symbols) and theoretical predictions (solid lines) of the fraction of formed DNA bonds as a function of temperature within (blue) and outside (green) the adhesion patch. Experimental values were extracted from *in situ* FRET spectroscopic measurements while theoretical curves were computed through an analytical framework coupling the statistical mechanics of DNA linkers to the deformability of vesicles. Shaded regions are propagated uncertainties, and the cyan region marks their overlap. Panels (b, bottom) and (c) adapted with permission from ref. 75 Copyright 2015, PCCP Owner Societies. (d) Fraction of adhering GUVs with respect to ligand/DNA receptor concentration ratio. Two regimes emerge in a re-entrant behaviour in which adhesion only takes place at intermediate concentration values, while at low and high values the GUVs cannot adhere due to a lack of stable bridging complexes or receptor saturation with ligands, respectively. Adapted with permission from ref. 77 Copyright 2017, American Chemical Society. (e) DNA-decorated GUVs can form both intra-membrane loops and inter-membrane bridges through 9-nucleotide stick-ends. Liposome pairs and networks sustain temperature-dependent morphological changes driven by the collective action of DNA interactions and the deformability of lipid membranes. At low temperatures the adhesion region (AR) shrinks resulting in a porous network, while the AR expands upon heating leading to no interstices. Adapted with permission from ref. 74 Copyright 2015, Macmillan Publishers Limited.

Systems of interacting GUVs and SLBs have been subsequently adopted to shed light on the statistical mechanical phenomenology of multivalent interactions between fluid membranes, thus paving the route to the rational design of artificial-cell adhesion and deepening our understanding of analogous biological processes. For their contribution, Shimobayashi *et al.* decorated both GUVs and SLB with two types of DNA receptors (A_1_ and A_2_) able to interact with each other *via* complementary sticky-ends, as shown in Fig. 2a.^75^ Thus, both intra-membrane loops and inter-membrane bridges could form, but only the latter would contribute to GUV–SLB adhesion. The authors proposed a statistical–mechanical model which could predict the probability of loop and bridge formation, alongside the resulting GUV–SLB adhesion free energy. The adopted experimental configuration (Fig. 2b), coupled to strategically-positioned cyanine fluorphores, allowed the authors to measure the temperature-dependent probability of bond formation thorough FRET, both inside and outside the GUV–SLB adhesion area (Fig. 2c). The experimental observation of a lower melting temperature for loop-dimers (present outside of the adhesion patch) matched the model predictions (Fig. 2c). The GUV–SLB configurations also allowed the authors to extract the membrane tension of the GUV through flickering spectroscopy.^121^ Also in this instance, comparison between experiments and the statistical–mechanical model revealed good agreement, both for the absolute value of the tension and its weak dependence on temperature.

Amjad *et al.* adopted a similar GUV–SLB system, but replaced the sticky ends on the DNA nanostructures with biotin modifications, so that adhesion would only be mediated by streptavidin. Indeed, in their implementation, membrane-bound DNA constructs featuring biotin moieties behaved as “receptors”, while streptavidin molecules assumed the role of “ligands”.^77^ Given the tetravalent nature of the latter, this design choice allowed them to explore the coupled role of ligand valency and ligand concentration on multivalent interactions. Liposome adhesion emerged only in a specific range of ligand/receptor concentration ratios, as shown in Fig. 2d, that is those ratios allowing for at least one receptor per bilayer to bind the same streptavidin moiety. This results in the formation of di- or multi-valent DNA–streptavidin complexes (*i.e.* two to four DNA receptors bound to the same streptavidin). At low ratios, streptavidin molecules were not numerous enough, resulting in too few DNA–streptavidin linkages to stabilise adhesion. Similarly, at very high concentration ratios, most of the streptavidin ligands would be bound to a single DNA receptor, thus also suppressing the formation of complexes featuring at least one receptor per bilayer. Only intermediate ligand/receptor ratios promoted the formation of complexes able to bridge the membranes and, therefore, sustain GUV–SLB adhesion. The work of Amjad *et al.* also showcased the promise that DNA–membrane systems hold for bioanalytical devices, where strategic design updates to target analytes of interest could pave the way for next-generation biosensing platforms. Indeed, one can envisage using the sharp onset of adhesion, observed upon reaching the analyte critical concentration, as a readout mechanism, for instance, by coupling it to optical or electrochemical measurements.

Beales and Vanderlick extended the application of multivalent DNA-mediated interactions from vesicle–SLB systems to the self-assembly of free-standing Large Unilamellar Vesicles (LUVs), exploring the effect of design and environmental parameters including surface coverage, ionic strength, temperature and membrane charge.^79,122^ With the aim of further enriching the available range of responses, Hernandez-Ainsa *et al.* demonstrated the light-controlled aggregation of LUVs, by utilising azobenzene motifs to tether the DNA linkers to the membranes.^123^ These photo-active moieties undergo conformational changes upon exposure to UV light, switching between *cis* and *trans* configurations characterised by different degrees of hydrophobicity. The transition thus results in a change in the affinity of the anchors for the bilayer, leading to light-triggered LUV aggregation and disassembly. Bachmann *et al.* combined experiments, coarse-grained computer simulations, and the aforementioned analytical framework for multivalent interactions to provide quantitative insight into the phase behaviour of DNA-functionalised LUVs.^76^ The authors focused on the effect of DNA-linker density on the melting temperature of the self-assembled phases and observed, intriguingly, that no aggregation occurs below a critical coverage owing to the competition between intra-LUV loops and inter-LUV bridges.^76^ This emergent phenomenon is enhanced by the lateral diffusivity of membrane-tethered linkers, which translates into an entropic advantage of loop over bridge formation, and could be rationally harnessed for the design of multivalent schemes for targeted drug delivery or artificial cell adhesion.^88^

Parolini *et al.* explored DNA-mediated multivalent interactions between cell-size GUVs, and conducted a comprehensive study of their adhesion and subsequent self-assembly into synthetic tissue-like materials.^74^ Using a functionalisation scheme similar to the one depicted in Fig. 2a, GUVs were decorated with receptors *A*_1_ and *A*_2_, enabling the formation of bridges and loops through mutual complementarity. Adhering GUV pairs and extended tissues were observed to have tuneable equilibrium features and temperature-dependent morphologies due to the coupling between the free energies of loop and bridge formation and the temperature-dependent excess area of the bilayer. In particular, the artificial tissues displayed an unusual thermal response, contracting upon heating and swelling on cooling (negative thermal expansion coefficient), as a consequence of changes in the extension of the GUV–GUV contact regions, and thus of the interstices present within the tissue (Fig. 2e). The observed behaviour is fully supported by the multivalent analytical framework mentioned above, where the temperature-dependent GUV area is accounted for. Such synthetic tissues pave the way for the construction of bio-compatible filtering devices and scaffolding platforms applicable in regenerative medicine.

**Fig. 3:**
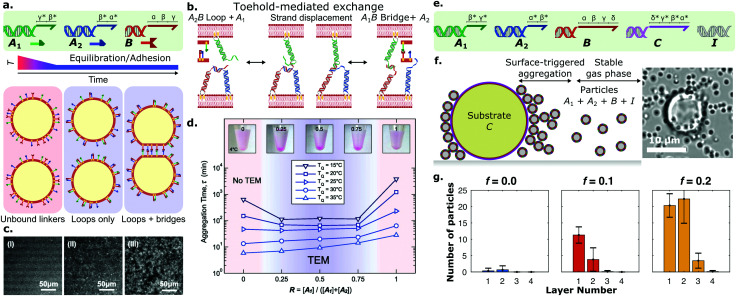
Programming the assembly kinetics of synthetic cellular systems with DNA receptors. Kinetic control over DNA interactions allows to program the isothermal self-assembly dynamics of cell-like objects. (a) Large unilamellar vesicles (LUVs) were functionalised with receptors *A*_1_, *A*_2_, and *B*. *B*, with a sticky-end featuring domains *α*, *β* and *γ*, can interact with *A*_1_ (featuring γ*β*) and *A*_2_ (β*α*) through complementarity. Heating the LUVs above the DNA complexation temperature (*T*_m_), followed by a rapid quench, induced a kinetically-arrested loop configuration (*i.e. A*_1_*B* or *A*_2_*B*), thus sequestering all *B* receptors available. While the emergence of inter-membrane bridges is thermodynamically favourable, the equilibration timescale is limited by the loop-to-bridge bond swapping. (b) Harnessing free domains *α* or *γ* in *A*_1_*B*(*A*_2_*B*) loops, a toeholding strand exchange mediates efficient bond swapping, catalysing the emergence of bridges and leading to LUV aggregation. (c) Representative epifluorescence micrographs following the aggregation kinetics of DNA-bearing LUVs when incubated at *T*_*Q*_ < *T*_m_, showcasing (i) early, (ii) intermediate, and (iii) late aggregation stages. Adapted with permission from ref. 81 Copyright 2016, American Chemical Society. (d) Aggregation half-times extracted *via* Fourier analysis of micrographs such as those in panel (c) at different relative abundances (*R* = [*A*_1_]/([*A*_1_] + [*A*_2_])) of *A*_1_ with respect to *A*_2_ and temperatures of incubation (*T*_*Q*_). Insets at the top are samples with increasing coating stoichiometries incubated at 4 °C for 2 days, where a toehold-mediated exchange mechanism (TEM) leads to aggregation and sedimentation. Adapted with permission from ref. 81 Copyright 2016, American Chemical Society. (e) A modified functionalisation scheme comprised of five DNA receptors: *A*_1_, *A*_2_, *B*, *C*, and *I*. Through sticky-end full or partial complementarity, *B* can interact with *C*, but also with *A*_1_ and *A*_2_. (f) (left) Silica substrate beads (∼10 μm) featured a supported lipid bilayer functionalised with *C*, while lipid bilayers on silica particles (∼1 μm) were decorated with *A*_1_, *A*_2_, *B*, and *I*. *I* was a long inert construct that provided colloidal stability. Particles thus featured intra-membrane *A*_1_*B*(*A*_2_*B*) loops. In the presence of the functional interface (*i.e. C*-bearing substrate bead), δ* toehold domains mediated a strand displacement reaction to form *BC* bridges, leading to adhesion of a first layer of particles onto the bead. In turn, the now free *A*_1_ and *A*_2_ receptors can interact with other particles and form inter-particle bridges *via* a toehold-exchange mechanism that leverages domains *α* or *γ*, triggering the growth and assembly of aggregates. The fraction of DNA receptors is defined as *f* = [*L*]/([*L*] + [*I*]), where [*L*] = [*A*_1_] + [*A*_2_] + [*I*]. (right) Bright-field micrograph of a representative substrate bead and surrounding particles featuring a fraction of receptors *f* = 0.2. Adapted with permission from ref. 109 Copyright 2020, The Royal Society of Chemistry. (g) Histograms conveying the average number of particles per adhesion layer at increasing fractions of receptors *f*. Adapted with permission from ref. 109 Copyright 2020, The Royal Society of Chemistry.

Besides providing fine control over the static properties of self-assembling synthetic cellular networks, multivalent DNA-mediated interactions also enable programming of adhesion kinetics. While the diffusion of liposomes and bilayer-anchored species are prescribed by the size, phase, and composition of the vesicles, the kinetics of DNA–DNA interactions can be engineered using well-established strand displacement mechanisms.^124,125^ This concept has been exploited by Parolini *et al.* to fine-tune the isothermal self-assembly of DNA-bearing liposomes into networks.^81^ As shown in Fig. 3a, LUVs featured a modified functionalisation scheme that consisted of three types of DNA receptors – *A*_1_, *A*_2_, and B – with [A_1_] + [A_2_] = 2[B]. Receptors *A*_1_ and *A*_2_ could interact with *B* but not with each other, again enabling the formation of *A*_1_*B* and *A*_2_*B* in both loop and bridge configurations.

A rapid thermal quench from high temperatures, where no bonds are favourable, resulted in the preferential formation of kinetically-favoured loops, sequestering the vast majority of *B* receptors, and thus preventing bridge formation and subsequent LUV aggregation (Fig. 3a). However, given that the thermodynamic equilibrium configuration displayed coexistence of loops and bridges,^88^ the system was bound to eventually relax to a state where (some) bridges are present, leading to liposome aggregation and tissue formation. In the absence of a catalytic driver to mediate the switching of bonds from loops to bridges, the equilibration would have to rely on thermally activated loop opening, which for the system in question would be prohibitively slow at room temperature owing to the relative stability of *A*_1_*B* and *A*_2_*B* interactions. The authors demonstrated how the bond-swapping kinetics, the emergence of bridges, and the subsequent formation of aggregates can be accelerated by a toehold-mediated exchange mechanism (TEM),^124^ as shown in Fig. 3b. Specifically, an unbound ssDNA domain available on *B* when forming a loop with *A*_1_(*A*_2_), offered a toehold for unpaired *A*_2_(*A*_1_) linkers on a nearby partner liposome, leading to bridge formation. The isothermal aggregation time of LUVs, extracted *via* Fourier analysis of fluorescence micrographs such as those shown in Fig. 3c, could be tuned over orders of magnitude by controlling the relative abundances of *A*_1_ and *A*_2_, which in turn impacted the availability of bond-swapping partners (Fig. 3d). Experimental trends were qualitatively replicated by a kinetic model which accounted for the (toeholding-modulated) rates of formation and breakup of two and three-linker DNA complexes.^126^ The ability of prescribing timescales of vesicle–vesicle adhesion onset could be invaluable for applications where precise control is required over the time-dependent mechanical properties of artificial tissues. For instance, one could envisage the design of synthetic cell formulations that can be injected *in vivo* while in liquid form, and programmed to readily set shortly afterwards to form a tissue implant.

Besides temporal control, multivalent DNA-mediated interactions can also be harnessed to program spatially heterogeneous self-assembly phenomena, as demonstrated by Lanfranco *et al.*^109^ Here, the authors exploited a toehold-mediated exchange interaction scheme similar to the one discussed above,^81^ and combined it with rationally-designed bond competition to demonstrate self-limiting colloidal self-assembly triggered by a functional interface. This concept, first demonstrated computationally by Jana and Mognetti,^127^ is reminiscent of several instances of biological self-assembly, where the occurrence and features of macro-molecular aggregates are finely regulated by their interactions with nearby interfaces. The experimental implementation of Lanfranco *et al.* consisted of bilayer coated silica substrates (large beads) and micron-size colloidal particles, both decorated by DNA linkers. The functionalisation scheme included five types of DNA nanostructures, as depicted in Fig. 3e: *A*_1_, *A*_2_, *B*, *C*, and *I*. Silica substrate beads featured receptors *C*, while particles were functionalised with constructs *A*_1_, *A*_2_, *B* and *I*. Receptors *A*_1_ and *A*_2_ could bind to *B*, but not each other, while *B* could also bind to *C*. Construct *I* was a long inert double-stranded nanostructure introduced to provide particle–particle steric repulsion. The latter prevented the formation of *A*_1_*B*(*A*_2_*B*) inter-particle bridges which would lead to spontaneous aggregation, resulting in turn in a stable colloidal gas phase. Here, most *A*_1_, *A*_2_ and *B* constructs were engaged in intra-particle loops, given that [*A*_1_] + [*A*_2_] = [*B*]. In the presence of the functional substrate, toehold domains in receptors *C* could catalyse the break-up of *A*_1_*B* or *A*_2_*B* loops, leading to particle adhesion onto the substrate *via* irreversible *BC* bridges. The sequestration of *B* linkers freed up initially saturated *A*_1_ and *A*_2_ constructs, shifting the thermodynamic equilibrium in favour of inter-particle bridges, whose formation kinetics was facilitated by toehold-mediated exchange as discussed above. This process led to the formation of a second particle layer onto the substrate, and could propagate to multiple layers in a self-limiting chain reaction, as depicted schematically and shown with a representative micrograph in Fig. 3f. Such a strategy showcased that the self-assembly and growth of the colloidal aggregate could be regulated by the presence of the functional substrate, while the final size of the aggregates could be controlled by fine-tuning the stoichiometries of receptors and inert constructs (Fig. 3g). Besides enabling spatially-coordinated colloidal self-assembly, this approach also provides a means of signal amplification in which the formation of large colloidal aggregates could report on the presence of biological analytes and biomarkers.

### 2.2 Beyond adhesion: biosensing platforms and membrane sculpting with DNA nanostructures

The functionalities of biological membrane machinery go well beyond controlling adhesion, as proteolipid interfaces are known to sustain and mediate critical responses such as sensing, communication, transport and morphological adaptation. Building onto the solutions developed for the “simple” sticky constructs discussed in the previous sections, several groups have exploited the functional versatility of amphiphilic DNA nanotechnology to replicate some of these capabilities in synthetic cellular systems. In this section we review some notable examples from our work and that of others, with particular emphasis on biosensing, membrane patterning and transport.

Cell membranes host receptors dedicated to transducing chemical signals and linking them to downstream signalling pathways. In some instances, such as for immunity-relevant toll-like receptors,^128^ signalling is triggered by analyte-mediated dimerisation of receptors. Kaufhold *et al.* took inspiration from this mechanism to implement a membrane-hosted biosensing platform reliant on target-induced DNA strand displacement (TIDSD).^82,129,130^ Here, the target analyte co-localises an invader DNA construct with a substrate-incumbent dimer, catalysing a strand displacement reaction similar to what happens for conventional toehold-mediated strand displacement.^129^ The authors demonstrated that compared to analogous circuitry freely diffusing in bulk, membrane hosted TIDSD showed up to a 2-fold increase in response rate, as confirmed with experiments and coarse-grained computer simulations.^131^ Moreover, they showed that the membrane scaffold helps reducing false positive signals, or leakage, a highly-coveted feature that could unlock their applicability in biosensing technologies.

Many of the functionalities mediated by cell-membrane receptors rely on a tight regulation of their lateral distribution on the plasma and internal membranes of the cells. While receptor complexation is to some extent responsible for lateral organisation, it is believed that preferential affinity for specific lipid micro-environments may also be critical. The notable example is that of lipid rafts, hypothesised to recruit membrane proteins and underpin processes like signal transduction, membrane trafficking, and lipid sorting.^132^ Combining amphiphilic DNA nanostructures with multi-component synthetic membranes displaying a rich phase behaviour^133^ enables biomimetic regulation of the lateral distribution, and consequently the functionality, of membrane inclusions.

**Fig. 4:**
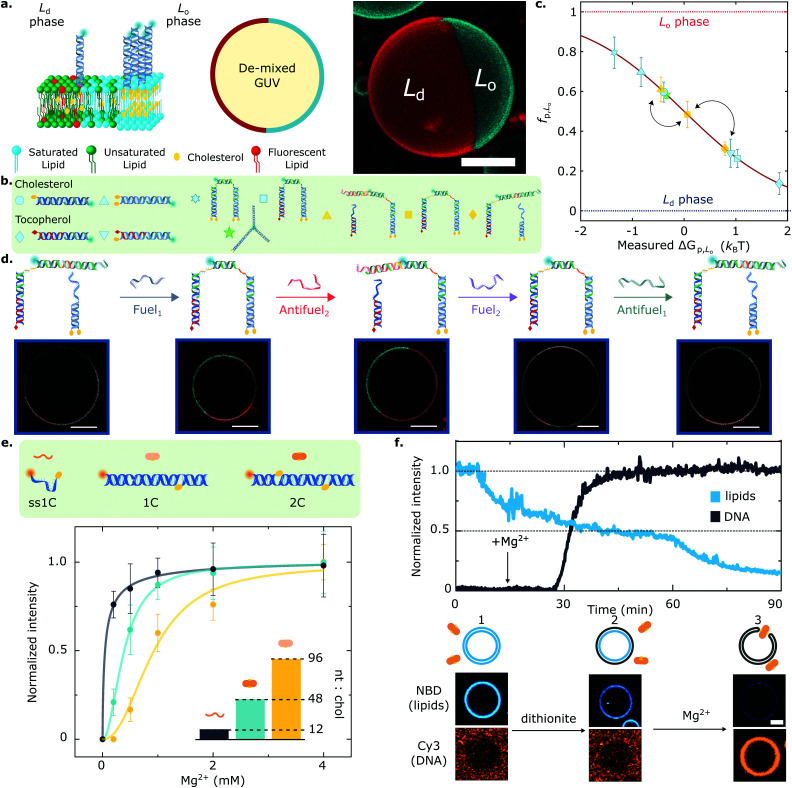
Lateral organisation and functional responses of membrane-bound DNA nanostructures. (a) (left) Giant Unilamellar Vesicles (GUVs) prepared from lipid mixtures of synthetic unsaturated and saturated lipids with cholesterol phase-separate into coexisting liquid ordered (*L*_o_) and liquid disordered (*L*_d_) phases. DNA nanostructures decorating the outer leaflet of de-mixed membranes undergo partitioning, as exemplified schematically with constructs bearing two cholesterol anchors that enrich the *L*_o_ domains. (right) 3D view from a confocal z-stack using a maximum projection view. DNA (cyan) partitions to the *L*_o_-phase, and the *L*_d_ domain is tagged with fluorescent TexasRed-DHPE (red). Scale bar = 10 μm. (b) The modularity of the platform allows to couple two or more anchors, starting from cholesterol and tocopherol moieties that target the *L*_o_ and *L*_d_ phase, respectively. (c) The rational combination of anchors enables programming partitioning behaviours that span the free energy landscape, as conveyed with mean ± standard deviation (std) of the fraction of DNA receptors in the *L*_o_-phase (*f*_p,*L*o_). Arrows connect the partitioning states achieved by the responsive DNA architecture described in panel (d). (d) Toehold-mediated strand displacement facilitates receptor re-configuration and cargo re-distribution by attaching or detaching a fluorescent oligonucleotide from tocopherol and double-cholesterol anchoring points. Representative confocal micrographs showcase the different partitioning states that each receptor configuration achieves. Scale bars = 10 μm. (e) Amphiphilic DNA nanostructures have a nucleotide-to-cholesterol (nt : chol) ratio (inset), a tug-of-war metric that can be exploited to fine-tune DNA–lipid complexation. Cations screen the surface charge of DNA constructs and zwitterionic membranes, thus enabling nanostructure anchoring. While higher nt:chol ratios need high cation concentrations to achieve a given membrane surface coverage (shown with Mg^2+^ ions), low nt:chol require less cations to achieve similar densities. (f) Lipid scrambling, mediated by a synthetic DNA enzyme (2C), is summarised with representative fluorescent traces of DNA and lipids (top) as well as micrographs (bottom). GUVs and DNA start in the absence of salt, where no attachment is observed (1). Upon the addition of reducing-agent dithionite, only the outer leaflet lipids undergo bleaching, resulting in ∼50% loss of fluorescence intensity (2). Adding Mg^2+^ ions mediates DNA nanostructure attachment, which spans the bilayer and connects the inner and outer leaflets, allowing the diffusion of inner lipids to the outer leaflet. Subsequently, these bleach when exposed to dithionite, leading to a further fluorescence decrease in the lipid signal (3) and confirming scramblase activity. Scale bar = 5 μm. Panels (a–d) are adapted with permission from ref. 83, and (e), (f) are adapted with permission from ref. 84.

Key for achieving this objective is the ability of amphiphilic DNA nanostructures to selectively enrich different (coexisting) lipid phases, depending on the chemical identity of the anchoring motifs,^134,135^ the lipid composition of the membrane,^80^ and the size of the nanostructures.^83,107^ Indeed, harnessing the preferential affinity that cholesterol and tocopherol moieties have for liquid-ordered (*L*_o_) and liquid-disordered (*L*_d_) lipid phases, respectively, Rubio-Sánchez *et al.* showed that the lateral distribution of membrane-tethered DNA nanostructures can be statically and dynamically programmed.^83^ In this contribution, DNA receptors were interfaced with GUVs displaying coexistence of *L*_o_ and *L*_d_ phases, each occupying a hemispherical domain and resulting in a Janus-like geometry, as shown in Fig. 4a. The synthetic DNA nanostructures were anchored to the bilayer *via* cholesterol, tocopherol, or combinations thereof (Fig. 4b), and were demonstrated to display a programmable tendency to distribute across the two domains, dependent on the number and chemical identity of the anchors. In particular, the authors demonstrated that the free energy change driving preferential partitioning is approximately additive in the contributions from each individual anchor featured in the construct. Non-additive effects were also observed for specific anchor combinations, membrane compositions and nanostructure design, notably including those induced by steric interactions between bulkier nanostructures. Thus, by prescribing anchor combination as well as changes to nanostructure size and topology, the partitioning of the DNA devices could be programmed to achieve several states that fully spanned the partitioning landscape, as shown in Fig. 4c.

Furthermore, the functionality of the platform was illustrated with a proof-of-concept biomimetic DNA architecture responsive to molecular cues. Exploiting toehold-mediated strand displacement, Fuel/Antifuel strands, capable of inducing re-configuration, enabled a fluorescent model cargo to attach or detach from anchoring points associated to distinct partitioning behaviours (Fig. 4d). The latter enabled the DNA receptors to reversibly transport the fluorescent cargoes across the surface of the vesicles by attaining programmed partitioned states. Importantly, the re-shuffling action of the DNA receptors, which evokes the recruitment of cell-surface entities, was observed to have characteristic re-distribution times of ∼5 min,^83^ which are comparable to that of biological machinery involved in T-cell activation^136^ and clathrin-mediated endocytosis,^103,137^ with equilibration timescales in the order of tens to hundreds of seconds.

Further adding to the arsenal of effects that one can exploit to program the response of DNA-decorated membranes, Morzy *et al.* unveiled the key role of cations in regulating the interactions between functional nucleic acid nanostructures and model bilayers.^84^ Combining experiments and atomistic simulations, they demonstrated that gel-phase zwitterionic membranes can bind unmodified DNA nanostructures thanks to the action of divalent cations bridging the anionic groups on the DNA and lipid head-groups. Interestingly, the attractive interactions did not occur for liquid-phase membranes, unlocking pathways to modulate membrane-DNA complexation through any of the external stimuli or design parameters that can influence lipid phase, including temperature and sterol content.^84^ Furthermore, Morzy *et al.* showed that cation concentration and chemical nature offer a handle to fine tune the affinity between hydrophobe-modified DNA constructs and liquid-phase membranes, modulating the competition between hydrophobicity-mediated attraction and Coulomb repulsion through charge screening. The authors found that the degree of screening required to trigger membrane attachment is dependent on a “tug-of-war” ratio between the number of negatively charged nucleotides and that of hydrophobic anchors in the constructs, as shown in Fig. 4e for a library of cholesterol-modified nanostructures. Morzy *et al.* then exploited this effect to reversibly trigger membrane attachment and activation of a DNA-based synthetic enzyme *via* the addition of magnesium. The synthetic “scramblase” enzyme, a device previously introduced by Ohmann *et al.*^42^ and later simplified by Sobota *et al.*,^43^ has the ability to catalyse exchange of lipids between the two membrane leaflets (scrambling) once bound to, and then inserted across, the bilayer. To demonstrate the cation-mediated activation of the enzyme, a fluorophore reduction assay was used in which the membranes were doped with lipids tagged with NBD, a molecule that is fluorescent in its oxidised state and undergoes bleaching upon reduction. The addition of membrane-impermeable reducing agent dithionite bleached NBD on the outher leaflet resulting in a ∼50% loss in fluorescence emission from the membrane (Fig. 4f). The addition of Mg^2+^ caused attachment and activation of the synthetic DNA enzyme. This in turn triggered transport of un-bleached NBD-tagged lipids to the outer leaflet, their exposure to dithionite, and a further decrease in fluorescence, thus demonstrating scramblase activity.

Indeed, as discussed in this section, amphiphilic DNA nanostructures show great promise to readily design and construct a vast array of devices capable of imparting and replicating functionalities associated to biological interfaces in artificial cellular membranes. Combining hydrophobes of different chemical identities and properties with the tuneable size, topology, and responsiveness of DNA architectures is key for engineering ever-more sophisticated and programmable biomimetic responses. We envisage that coupling (amphiphilic) DNA nanotechnology with model membranes will open up a breadth of avenues for artificial cell science, and revolutionise the state-of-the-art in bottom-up synthetic biology.

## 3 Bulk self-assembly of amphiphilic DNA nanostructures

Besides enabling prescribable interactions with lipid membranes, the functionalisation of DNA nanostructures with hydrophobic moieties, ranging from small molecules to dendrons and polymers,^138^ has been exploited to enhance their self-assembly capabilities beyond what is achievable by base-pairing.

While base-pairing offers exquisite control over interaction strength and selectivity, its rigid “lock-and-key” nature means that programming higher-order self-assembly of the nanostructures requires very precise geometrical and thermodynamic optimisation.^139–142^ The attachment of hydrophobic tags provides DNA nanostructures with an amphiphilic character, granting access to self-assembly pathways where the thermodynamic ground state can be determined through the size, shape and topology of the DNA amphiphilies – all features that can be easily and robustly prescribed.^143–147^ While amphiphilic self-assembly principles do not allow for the molecular-scale localisation precision of base-paring, they are agnostic to the fine details of the nanostructures, which makes them robust against polydispersity and small design variations. In addition, the amphiphilic character of the resulting hydrophobised-DNA phases makes them ideal for programming interactions with other biological macromolecules and lipophilic small molecules, a useful characteristic for several applications including drug delivery and – relevant to the present discussion – the construction of biomimetic systems. In this section we review instances in which the amphiphilic self-assembly principle has been applied to programming higher-order self-assembly of DNA nanostructures. We largely focus on multi-functional nano-porous phases developed by our group in recent years, and comment on their potential application in bottom-up synthetic biology as well as other contexts.

Among the first examples of complex objects self-assembled from amphipilic DNA nanostructures are the DNA-somes, developed by Luo and coworkers.^148^ These particles, formed from lipid-modified DNA junctions could be tuned in size and have been shown to aid intra-cellular delivery of miRNA. Alberts *et al.* have later developed their version of DNA-somes, which thanks to pH-responsive i-motifs were able to switch between networks and vesicle-like objects, and may represent interesting and highly-programmable alternatives to lipid or polymer-based membranes for artificial cell implementations.^149^ Another notable example is the one demonstrated by Sleiman and coworkers, who explored the use of hydrophobic polymer^150,151^ or dendrite^152^ moieties to guide the self-assembly of nanocages in unique architectures, including intra or inter-molecular micellar assemblies capable of loading a hydrophobic cargo.

**Fig. 5:**
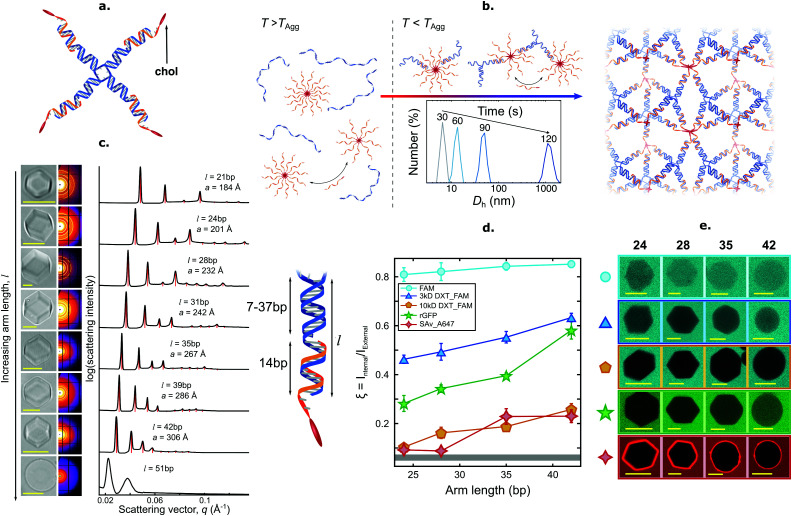
Self-assembly of amphiphilic DNA C-Stars into frameworks with tunable porosity. (a) C-Stars are comprised of four different core strands (blue) dictating the nanostar shape, and four cholesterolised strands (orange) granting amphiphilicity. (b) Schematic of self-assembly mechanism for C-Stars. A stoichiometric mixture of cholesterolised and core strands is heated and then slowly annealed. Above the melting temperature of the duplex arms (left) the mixture comprises both cholesterol-induced micelles and single-stranded core-forming oligonucleotides. Upon cooling below *T*_Agg_ = 77.1 ± 0.2 °C (centre), the core nanostar motifs start forming *via* hybridisation, thus bridging the micelles. DLS measurements (inset) demonstrate the nucleation and growth of aggregates over time, as exemplified by the increase in hydrodynamic diameter *D*_h_, eventually leading to extended crystalline frameworks (right). (c) Controlling arm length allows for lattice parameter tunability. Bright-field micrographs (left) depict rhombic-dodecahedral crystallites for C-Stars with *l* = 21–42 bp, while *l* = 51 bp produces amorphous spherical aggregates. SAXS powder diffraction patterns and the derived radially averaged profiles (centre) confirm BCC crystalline phase of C-Star aggregates for *l* = 21–42 bp and amorphous nature for *l* = 51 bp. Red vertical lines illustrate best fit to Bragg peaks for BCC symmetry. (right) Schematic illustrating the mechanism behind arm length control. Lattice parameter tunability ensures controllable porosity, thus enabling the use of C-Star frameworks as macromolecular sieves. (d) Porosity was assessed *via* permeation assays with a range of fluorescent probes: sodium fluorescein (FAM), fluorescein-labelled 3 kDa dextran (3 kDa DXT-FAM), fluorescein-labelled 10 kDa dextran (10 kDa DXT-FAM), recombinant GFP (rGFP) and Alexa647-labelled streptavidin (SAv-A647). The ratio *ξ* between average fluorescence intensity (from confocal micrographs) inside single crystals and in the surrounding probe-rich solution was used as proxy for probe permeation and partitioning. Gray band at the bottom depicts background fluorescence intensity. (e) Representative confocal micrographs corresponding to the data in (d). All scale bars = 10 μm. Panels (a–e) are adapted with permission from ref. 90. Copyright 2018, American Chemical Society. Panel (b) is adapted with permission from ref. 89. Copyright 2017, American Chemical Society.

Brady *et al.* introduced a versatile approach to the self-assembly of nano-porous, functional DNA phases reliant on cholesterolised nanostructures dubbed C-Stars.^89–91^ As shown in Fig. 5, these are simple DNA junctions featuring (typically) four dsDNA arms. However, rather than terminating in ssDNA sticky ends, as initially proposed by Seeman^1,153^ and later implemented by multiple groups,^3,154–163^ the arms are tipped by a cholesterol moiety, which confers an amphiphilic character to the nanostructures and drives their self-assembly into extended frameworks. Cholesterolised and non-cholesterolised ssDNA components were stoichiometrically mixed, and then slowly cooled from a high temperature – above the melting temperature of any duplex present. Upon cooling, the designed dsDNA motifs formed and started cross-linking existing DNA–cholesterol micelles, until a phase transition was encountered. At this stage aggregates nucleated in the bulk and started to coalesce and grow, ultimately forming an extended network (Fig. 5b). The resulting aggregates were shown to have an amorphous character if quenching was rapid, or form crystalline phases if annealed at a slow rate, as determined through SAXS and microscopy (Fig. 5c). C-Star single crystals were observed to exceed 40 μm in size, and the lattice parameter of the cubic unit cell could be finely programmed by changing arm length, spanning a range between ∼18 and ∼34 nm.^90^ Such a difference in lattice parameter directly translated into a difference in the network pore size, thus allowing C-Star aggregates to behave as controllable molecular sieves, where certain macromolecules can permeate and others cannot due to their bulky size. The authors performed permeation assays on various probes to confirm this behaviour, as summarised in Fig. 5d and e. Small molecules such as sodium fuorescein easily permeated even the lowest-porosity frameworks, while bulkier dextrans displayed a marked permeation increase upon increasing arm length. Notably, the emptiest crystalline frameworks achieved a free volume fraction in excess of 85%, comparable with ultra-high porosity metall–organic frameworks,^90^ thus confirming the potential of C-Star frameworks for cargo-loading applications, as required, for instance, in drug delivery applications.

**Fig. 6:**
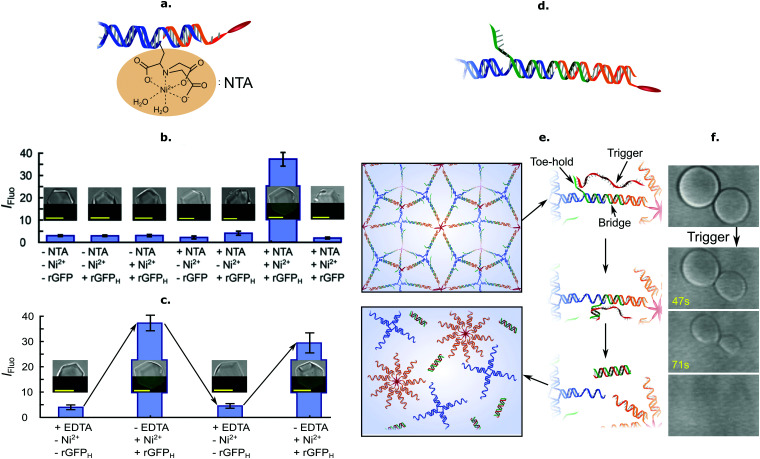
Functional frameworks of amphiphilic DNA nanostructures. (a) Inclusion of a nitrilotriacetic acid (NTA) functional group *via* chemical linking allows for specific and reversible protein entrapment. (b) Specific entrapment of N-terminal 6× histidine tagged rGFP (rGFP_H_) in NTA-functionalised single crystals (+NTA) in the presence of Ni^2+^ ions (+Ni^2+^). Lack of either the NTA functional group (−NTA, equivalent to pristine crystals), the histidine tags defining the target protein (rGFP *vs.* rGFP_H_) or Ni^2+^ ions (−Ni^2+^) fails to produce binding. Entrapment is assessed *via* confocal microscopy, by evaluating the mean fluorescence intensity inside single crystals, *I*_Fluo_. Insets depict composite bright-field (top) and fluorescence (bottom) images corresponding to the various investigated conditions. (c) Reversibility of target protein (rGFP_H_) entrapment in NTA-functionalised frameworks is achieved by cycling of Ni^2+^ concentration in solution. The availability of Ni^2+^ ions is increased by addition and reduced by chelation *via* EDTA. Insets follow the same rationale as for panel (b). (d) Inclusion of a toehold-bearing bridge strand linking core and cholesterol strands enables isothermal framework disassembly by toehold-mediated strand displacement. (e) Schematic of the strand–displacement mechanism leading to aggregate disassembly. Addition of an invading trigger strand (red), fully complementary to the bridge strand (green), leads to dissociation of cholesterol strand micelles from the nanostar motifs and melting of the aggregates. (f) Bright-field micrographs depicting rapid disassembly after addition of trigger strand. All scale bars = 10 μm. All panels adapted with permission from ref. 90. Copyright 2018, American Chemical Society.

The facile functionalisation of DNA nanotructures further allowed Brady *et al.* to make the cargo-loading ability of C-Star frameworks selective and stimuli-responsive.^90^ To this end, the authors functionalised individual C-Stars with a nitrilotriacetic acid (NTA) group, as shown in Fig. 6a, which is capable of selectively binding His-tagged proteins in the presence of Ni^2+^ ions. The authors applied this system to demonstrate selective trapping of His-tagged recombinant Green Fluorescent Protein (rGFP) (Fig. 6b), and its reversible release upon Ni^2+^ chelation *via* EDTA (Fig. 6c).

Besides releasing pre-loaded cargoes upon exposure to external cues, frameworks of amphiphilic DNA junctions were also shown to structurally respond to various stimuli. For instance, Brady *et al.* implemented a design change to the basic C-Star architectures, shown in Fig. 6d, to make the crystalline phases able to disassemble in the presence of a trigger ssDNA strand. The scheme relied on a toeholding reaction, through which the trigger sequestered a bridge strand initially linking the cholesterolised moiety to the nanostar core, thus leading to isothermal melting of the aggregates (Fig. 6e and f).

To further expand the range of stimuli to which amphiphilic DNA frameworks are able to respond, Fabrini *et al.* recently developed a strategy to render the materials structurally susceptible to changes in the identity and concentration of cations in solution.^164^ To achieve such functionality, the authors replaced the central junction in C-Stars with a tetramolecular DNA G-quadruplex (G4), a non-canonical motif formed in guanine-rich nucleic acids and stabilised by alkali metal ions.^165,166^ By exploiting the different degrees of G4 stabilisation offered by strongly-promoting K^+^ ions and other metal ions (Li^+^, Mg^2+^), the modified amphiphilic nanostars, dubbed Quad-Stars, displayed the sought sensitivity towards cation identity and concentration. The authors demonstrated that self-assembly rates depend steeply on K^+^ concentrations and the length of the G4-forming guanine runs. The frameworks could be quickly disassembled by chelating K^+^, and thus release previously loaded cargoes. Finally, the inclusion of a photosensitising G4-binding porphyrin enabled the light-induced (irreversible) disassembly of the hydrogel aggregates, adding yet another route to trigger structural responses in the amphiphilic DNA frameworks.

While such *ad hoc* design modifications can lead to de-stabilisation of the amphiphilic DNA frameworks in response to changes in the ionic conditions, “conventional” C-Star designs have been shown to remain stable under a broad range of ionic strength and, importantly, at physiological values (1× phosphate-buffered saline, PBS).^91^ This characteristic is key for foreseen biomimetic applications, and gives amphiphilic DNA building blocks an advantage over other network-forming DNA motifs (including small nanostructures^139^ and origami^167–169^), whose stability has been reported to rely on the availability of divalent cations.^170^ While, indeed, some DNA origami have been shown to remain stable even in low-Mg^2+^ buffers,^171,172^ structural integrity heavily depends on topological complexity,^171,173,174^ with more intricate 3D architectures or networks^175^ requiring either divalent cations or extremely high (non-physiological) concentrations of monovalent salts.^173^ It should however be noted that depending on C-Star design, cation identity has been found to impact the emergence of crystalline as opposed to amorphous phases, as systematically investigated in ref. 91. The effect is ascribed to the ability of divalent cations, and in particular magnesium, to stabilise a stacked configuration of the central DNA junction, whose rigid geometry was hypothesised to disrupt the crystalline structure.

While the initial contributions by Brady *et al.*^89–91^ and later Fabrini *et al.*^164^ have demonstrated a remarkable ability to program stimuli-responsiveness and nanoscale structural properties of amphiphilic DNA phases, many potential applications of these materials would benefit from precise control over the size of the aggregates themselves. This is the case, for instance, of intra-cellular cargo delivery that would require vectors in the hundred-nanometre range for optimal uptake.^176,177^ To improve on this aspect, Walczak *et al.* devised a strategy which enables the production of amphipilic DNA aggregates with sizes ranging from a few hundred nanometers to several microns.^178^ Specifically, the authors adopted a two-step self-assembly protocol in which an initial rapid quench from high to intermediate temperature leads to C-Star aggregate nucleation and initial growth, and is followed by a second quench from intermediate to low temperature which triggers the passivation of the aggregates with a non-sticky (hydrophilic) DNA corona. The latter arrests the growth of the amphiphilic DNA aggregates, whose size can thus be programmed by tuning the incubation time at intermediate temperature. The particles were found to remain stable over extended periods of time, and could be modified to displace the protective corona upon external stimulation. Corona displacement led to exposure of the amphiphilic core of the particles, triggering their assembly into “sticky” gel-like aggregates able to disrupt lipid membranes and capture swimming bacteria. The latter ability is reminiscent of that of killer T-cells to secrete sticky networks made of their own DNA to trap pathogens.^179–182^

The amphiphilic DNA frameworks reviewed in this section have been demonstrated to possess an array of unique features, including controllable porosity,^90^ responsiveness to various molecular and environmental stimuli,^90,164^ programmable aggregate size,^178^ and triggered interactions with lipid membranes and living cells.^178^ These characteristics make them potential candidates as both structural and functional elements in artificial cell implementations. As proposed and demonstrated in several instances, membrane-less compartments^64,65^ represent an intriguing alternative to membrane-based enclosures to serve as scaffold for artificial cells. Examples reported to date have been often reliant on various forms of polymer coacervates or hydrogels,^59–63^ which, while enabling a degree on control on composition and molecular partitioning, offer little opportunity for the rational design of life-like responses in which structural properties are coupled to molecular pathways. In turn, thanks to the programmability of the nucleic acid building blocks, amphiphilic DNA scaffolds open up new opportunities for co-engineering structural and information-processing aspects of artificial cells. One could indeed envisage implementations in which communication/sensing pathways, hosted by the DNA building block themselves, could lead to structural changes in the network, thus impacting porosity and molecular transport, and ultimately feeding-back to the artificial signalling mechanism. In addition, the structure–function synergy unlocked by the responsive all-DNA scaffolds could lead to programming cell-like behaviours requiring regulated changes to global morphology, such as uptake of large objects, growth, and division which have been traditionally challenging to implement.^58,63^ Finally we note that amphiphilic DNA frameworks may also serve as functional internal compartments, or membrane-less organelles in membrane-based artificial cell implementations, where they could lead to spatial localisation and segregation of functionalities – a critical step towards the construction of intricate signalling pathways such as those observed in eukaryotes.^57^

## 4 Conclusive remarks and future challenges

As a feature article centred around the work of our group, this contribution is not intended to offer a comprehensive snapshot of the rapidly growing field of biomimetic DNA nanotechnology, nor of the substantial sub-set of studies specifically addressing challenges in bottom-up synthetic biology and artificial cell science. We have, for instance, omitted discussion of key contributions on the use of DNA origami to control mechanical properties of synthetic bilayers^85–87^ or to regulate transport across them by establishing transmembrane pores,^183,184^ as well as notable efforts to program communication using DNA-based molecular circuitry.^27^ The powerful capabilities highlighted in these contributions, alongside the ones discussed in more detail here, print an exciting picture for the near future of this research area. In the remainder of this section we will highlight aspects that, in our opinion, deserve particular attention from the community.

The first concept we would like to explore is that of integration. One of the key advantages of bottom-up synthetic biology is the availability of a virtually unlimited library of components and mechanisms, both biological and inorganic, that one can draw from when designing and constructing an artificial cell. This has resulted in a substantial heterogeneity across implementations designed to display different behaviours, which makes challenging to integrate them in multi-functional artificial cells. In other words, while examples of artificial cells capable of either sensing, moving, communicating, or dividing (to mention a few examples) have been extensively demonstrated, we are still unable to construct an artificial cell that can simultaneously display and coordinate many of those behaviours because the solutions developed for each are incompatible. Synthetic DNA-nanosystems, owing to the chemical and functional homogeneity of their constituents, could help overcoming this critical limitation. It is indeed easy to envisage how biomimetic DNA nanostructures designed separately to regulate transport, adhesion, communication and sensing could be interfaced with each other, and coupled to common signalling pathways reliant on DNA-based molecular circuitry. Success in the holistic design of these “all-DNA” artificial cells, with integrated control of multiple behaviours, could take the field one step closer to the construction of truly cell-like micro-robots.

The second aspect we would like to discuss is that of free energy regeneration. Living systems are sustained by a constant free energy flux, which keeps them hovering above thermodynamic equilibrium. Free energy is sourced from the environment and then distributed or stored in the form of chemical vectors. Instead, for the most part, dynamic DNA nano-systems are driven by discrete relaxations from initial high-free-energy states. The latter are typically characterised by fewer base-pairing bonds compared to the thermodynamic ground state, and can be made kinetically meta-stable, so that the free energy tap can be opened only in the presence of a specific trigger. While remarkable design advances have been recently introduced, which enable decoupling thermodynamic drive from kinetic rates^185^ and systematically accessing out-of-equilibrium configurations,^186^ these free-energy reservoirs remain essentially non renewable, limiting their applicability to the construction of autonomous agents. There is therefore a need for solutions that can continuously regenerate free energy reservoirs in DNA nano-systems from readily available environmental sources. Thankfully, examples are emerging demonstrating the coupling of nucleic-acid molecular reaction networks and self-assembly to enzymatic processes,^187–191^ also in the context of cytomimetic agents.^192^ Given these encouraging results, we argue that a generalised enzymatic pathway for the sustained production of a nucleic acid “free energy currency” would be a welcome development for the community, and unlock the modular design of self-sustaining, DNA-based artificial cells.

## Conflicts of interest

There are no conflicts to declare.

## Supplementary Material
